# Iron-Palladium Decorated Carbon Nanotubes Achieve Radiosensitization via Reactive Oxygen Species Burst

**DOI:** 10.3389/fbioe.2021.683363

**Published:** 2021-05-21

**Authors:** Shengnan Yang, Yiling Yang, Yi Yang, Xiangya Zhao, Qian Wang, Bing Li, Ling Dong, Rui Tian, Zhirong Bao

**Affiliations:** ^1^Department of Geriatric Medicine, The First Affiliated Hospital of Zhengzhou University, Zhengzhou, China; ^2^Department of Ultrasound, The First Affiliated Hospital of Zhengzhou University, Zhengzhou, China; ^3^Hubei Key Laboratory of Tumor Biological Behaviors, Department of Radiation and Medical Oncology, Hubei Cancer Clinical Study Center, Zhongnan Hospital of Wuhan University, Wuhan, China

**Keywords:** FePd NPs, carbon nanotubes, nanomedicine, radiotherapy, retention

## Abstract

Radiotherapy is recommended as a modality for cancer treatment in clinic. However, cancerous cells were resistant to therapeutic irradiation due to its DNA repair. In this work, single-walled carbon nanotubes with unique physical properties of hollow structures and high specific surface area were introduced as carrier for iron-palladium (FePd) to obtain iron-palladium decorated carbon nanotubes (FePd@CNTs). On one hand, FePd nanoparticles possess significant ability in radiosensitization as previously reported. On the other hand, carbon nanotubes offer higher efficiency in crossing biological barriers, inducing the accumulation and retention of FePd nanoparticles within tumor tissue. In order to verify the radiosensitization effect of FePd@CNTs, both *in vitro* and *in vivo* experiments were conducted. These experiments showed that the FePd@CNTs exhibited remarkably better radiosensitization effect and more obvious accumulation than FePd NPs, suggesting a potential of FePd@CNTs in radiosensitization.

## Highlights

- Iron-palladium decorated carbon nanotubes (FePd@CNTs) were synthesized.
- FePd@CNTs could induce reactive oxygen species (ROS).
- FePd@CNTs exhibited a prolonged circulation time.

## Introduction

Radiotherapy is a cancer therapy method that uses radiation rays to kill tumor cells directly or indirectly. More than 60% of cancer patients receive radiotherapy during the treatment (Schaue and McBride, 2015; Sasieni and Sawyer, 2021). Radiation leads to DNA double strand breaks (DSBs), thereby causing apoptosis of cancer cells. Though radiotherapy has been widely used in cancer treatment in clinical, there are still many problems to be solved. Firstly, radiation rays would inevitably damage normal tissues and organs while ablate tumor tissue due to the inability to differentiate tumor cells from normal cells, thus leading acute and late side effects (Gudkov and Komarova, 2010). In addition, radiation resistance caused of cancerous cells induced by the hypoxic in tumor tissues is another barrier that impairs the effectiveness of radiotherapy (Carlson et al., 2006; Stewart et al., 2011). Reactive oxygen species (ROS) in cells lead to apoptosis (Hirayama et al., 2009; Yang et al., 2017). But it is reported that ROS levels in some tumors are lower than corresponding non-tumorigenic cells (Diehn et al., 2009). Hence, two main strategies appears to be the key to solve these problems: (1) accurate tumor delineation and image guidance technologies with higher resolution (Bhide and Nutting, 2010); (2) high-efficiency radiosensitizers to specifically increase the radiation deposition in tumors (Zhu et al., 2020a), reduce tumor hypoxia (Lyu et al., 2020b) and increase the production of reactive oxygen species in tumor cells (Liu et al., 2020).

In recent years, various nanomaterials have emerged as radiosensitizers. Nanomaterials with high atomic number (Z) elements is capable of enhancing the photoelectric effect and Compton effect of radiation rays as well as improving the effective deposition of rays. Therefore, high-Z elements such as gold (Xia et al., 2020; Zhu et al., 2020a), silver (He et al., 2020; Khochaiche et al., 2021), platinum (Luo et al., 2020) and other nanoparticles (Deng et al., 2018) have been extensively explored as radiosensitizers. The tumor microenvironment (TME) is abundent in hydrogen peroxide (Balkwill et al., 2012; Taddei et al., 2013; Chen et al., 2017; Lyu et al., 2020b). Thus, nanoparticles containing manganese dioxide which could catalyze hydrogen peroxide to generate oxygen was used for alleviating hypoxia in tumors regions (Abbasi et al., 2016; Yi et al., 2016) and hence fix the DSBs in cells after radiotherapy. With a high specific surface area, some nanoplatforms provide possibility for functional surface modification and hence could be used as nanocarriers for radiosensitizers and anticancer drugs through endocytosis (Lu et al., 2018; Zhu et al., 2020b). When triggered by X-rays or the special microenvironment in tumor (mild acidity, over expressed H_2_O_2_, etc.), these nanocarriers could release drugs, offering an outstanding targeting effect (Wang et al., 2014). In addition, nanomaterials are widely used for the combination of radiotherapy and other tumor treatment methods to enhance the therapy outcome such as radiochemotherapy (Xiong et al., 2015), X-rays induced photodynamic therapy (Wang et al., 2016), thermoradiotherapy (Lyu et al., 2020a). Meanwhile, nanomaterials which both radiotherapy enhancing property and imaging improving ability could also be an agent for image guided radiation therapy (IGRT) (Jølck et al., 2015). Both developing multifunctional nanostructures for enhancing the effect of therapy and improving drug delivery by nanocarrier appear to be significant methods for cancer therapy. Despite of the mentioned above advantages, problems still exist when nanoparticles are applied in radiotherapy, such as poor biocompatibility (Xia et al., 2020) and short circulation time (Sun et al., 2020) *in vivo* due to their ultra-small size (Dou et al., 2016; Luo et al., 2019).

**SCHEME 1:**
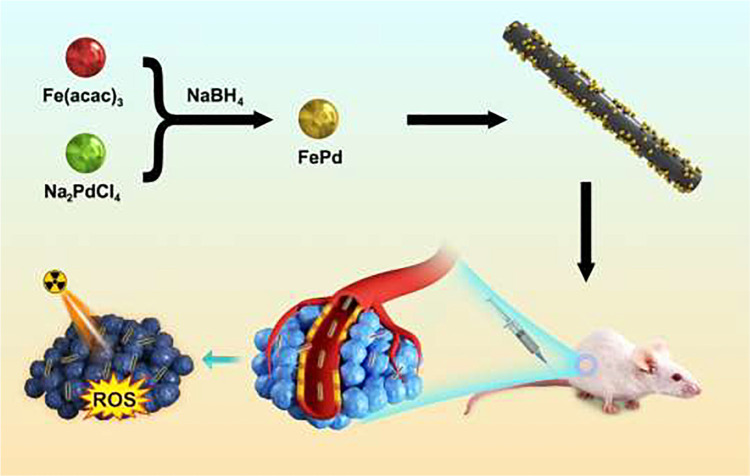
Synthesis of FePd@CNTs and radiotherapy enhancing mechanism.

FePd nanoparticles (NPs) enhance radiotherapy via NIR-II photothermal therapy as previously reported (Lyu et al., 2020a). Fenton reaction occurs when Fe react with rich hydrogen peroxide in the tumor, producing a large amount of ROS which leads to apoptosis (Ranji-Burachaloo et al., 2018). Meanwhile, Pd can improve the effective deposition of radiation. Therefore, ultra-small FePd NPs are remarkable radiosensitizers. Carbon nanotubes are widely used as nanocarriers for nanomedicine delivery (Feazell et al., 2007; Wong et al., 2013). They possess good biocompatibility and therefore could be more easily assimilated by cancer cells (Kostarelos et al., 2007). In this study, we loaded FePd NPs on carbon nanotubes to design a more effective nanostructure FePd@CNTs for achieving better radiosensitization effect (Scheme 1). This nanomaterial can be passively target into tumor tissues through the enhanced permeability and retention (EPR) effect (Fang et al., 2011; Maeda, 2017). Carbon nanotubes serve as carriers to deliver FePd NPs into the cells, which provide possibility for FePd NPs to deposit more radiation energy and produce abundant ROS to induce tumor cells apoptosis. In addition, compared with bare FePd NPs, using carbon nanotubes as a carrier could improve the drug circulation time *in vivo*, thus achieving the purpose for one injection but multiple radiotherapy enhancing effect during the whole treatment course. *In vitro* experiments including cell cytotoxicity using CCK 8 kit, live-dead staining and clonogenic assay of cells demonstrated that the FePd@CNTs possess satisfied biocompatibility and significant radiosensitization effect. The pharmacokinetic analysis and antitumor study on mice proved that FePd@CNTs could prolong the circulation time *in vivo* and achieve the therapeutic effect we expected. Tumor section staining in various treatment groups proved that the material induced tumor cell apoptosis by increasing the production of ROS in the tumor, thereby inhibiting tumor cell proliferation. Hence, using carbon nanotubes as a carrier can improve the radiosensitivity effectiveness of FePd NPs.

## Materials and Methods

### Materials and Characterization

Single-walled carbon nanotubes were purchased from Hengqiu Graphene Co., Ltd. (Suzhou, China) Iron (III) 2,4-pentanedionate (C_15_H_21_FeO_6_), palladium (II) sodium chloride (Na_2_PdCl_4_), oleylamine and polyvinylpyrrolidone (PVP, Mw = 10,000) were purchased from Aladdin Reagent Co., Ltd. (Shanghai, China). Sodium borohydride (NaBH4) was purchased from Lingfeng Chemical Reagent Co., Ltd. (Shanghai, China). Oleic acid and absolute ethanol were purchased from Sinopharm Chemical Reagent Co., Ltd. (Shanghai, China).

### Characterization Methods

The morphology was obtained in JEM-2010 FEF (JEOL, Japan) transmission electron microscope (TEM). Phase structures were obtained by Powder X-ray diffraction (XRD) using D8 Advance (Bruker, Germany) at a scanning rate at 2 ° per minute, 2θ range from 5° to 90°. The surface chemical elements of the samples were analyzed by X-ray photoelectron spectroscopy (XPS) with ESCA-LAB250Xi (Thermo, United States).

### Synthesis of FePd NPs and FePd@CNTs

FePd NPs were synthesized by reported method (Lyu et al., 2020a). A chemical reduction method was used to synthesized FePd@CNTs. 0.12 mg Fe(acac)_3_, 0.1 g Na_2_PdCl_4_,1.5 mL oleylamine and 1.5 mL oleic acid were successively dissolved in anhydrous ethanol in a round-bottom flask, followed by magnetic stirring for 30 min. Then, 25 mg carbon nanotubes and 5 mg PVP were dispersed in 10 mL anhydrous ethanol by sonication and added dropwise in the round-bottom flask. Next, excess ethanol solution of NaBH_4_ was added dropwise into the mixture. The mixture was stirred for 2 h at 40°C. Finally, products were separated by centrifugation, dried in a vacuum oven overnight and stored in a desiccator for further research. For synthesis of nanomaterials with various ratio of palladium and iron, the ratio of precursor of palladium and gold was monitored. For Fe:Pd = 3, 0.36 mg Fe (acac)_3_ and 0.1 g Na_2_PdCl_4_ were added.

### Cell Culture

The MCF-7 cell lines were assessed from the Cell Bank of the Chinese Academy of Sciences. Cells were incubated in DMEM high glucose medium with 10% FBS in an incubator (Sanyo, Japan) at 37°C with 5% CO_2_.

### Cytotoxicity Assay

The biocompatibility was evaluated by CCK 8 assay. The MCF-7 cells were seeded in 96-well plates (5 × 10^4^ cells per well), five wells per group. After being cultured for 24 h, the cells were treated with different concentrations (0, 10, 20, 50, 100, 200, and 500 μg/mL) of FePd NPs or FePd@CNTs for 24 h. The cells were incubated for 2 h after being added 10 μl CCK8 assay solution, then the absorbance value at the characteristic peak of 450 nm was evaluated by microplate reader (Rayto-6000 system, Rayto, China).

### Live/Dead Staining Assay

Live/dead staining experiment was carried out to assess cell viability. MCF-7 cells were seeded in six-well plates. Cultured for 24 h, the cells were treated in six groups: (i) Control, (ii) FePd NPs, (iii) FePd@CNTs, (iv) RT, (v) FePd++RT, and (vi) FePd@CNTs+RT, the concentration of FePd NPs and FePd@CNTs is 200 μg/mL, the dose of RT is 6 Gy. After corresponding treatment for 24 h, the cells were incubated with Calcein-AM/PI solution (4 μM for each fluorescent probe in PBS) for 1 h. The living (green) and dead (red) cells were observed using a fluorescence microscopy (Olympus IX 73 DP80, Japan) and photographed, then the fluorescence intensity were calculated using ImageJ 1.52 u software.

### Colony Formation Assay

The radiation sensitivity of FePd@CNTs was evaluated using a colony formation assay. Viable MCF-7 cells (100, 200, 300, 400, and 500 cells per well) were seeded in six-well plates. Once cell adhered, the cells were treated by (i) Control (no nanodrugs), (ii) FePd NPs (200 μg/mL), and (iii) FePd@CNTs (200 μg/mL), then received irradiation with 0, 2, 4, 6, and 8 Gy (three wells per group) by the Small Animal Irradiator (PXI X-RAD 225 Cx, North Branford, CT, United States). After 15 days, colonies were fixed using 4% paraformaldehyde and stained with crystal violet. The numbers of colonies were counted, then fit the survival curve using the “multi-target single-hit model.”

### Cellular Uptake

The cellular uptake of FePd@CNTs and FePd NPs was directly observed by TEM. MCF-7 cells were seeded in six-well plates and incubated with 200 μg/mL FePd NPs or FePd@CNTs for 24 h. Then craped the cells with cell scraper and fixed them with fixation for electron microscopy. Afterward, a series of dehydration were performed and the cells were then embedded and sliced into sections, followed by double staining with lead and uranium. Finally, observed the sections using a transmission electron microscopy (HT 7700, Hitachi, Japan).

### Animal Model

Female, 6 weeks old BALB/c mice were acquired from Vital River Company (Beijing, China). They were reared in a specific pathogen-free, temperature and humidity-controlled environment with clean food and water in their cages. To create tumor-bearing mice, 100 μL of 4T1 cell suspension (1 × 10^7^ cell per mL) were subcutaneously transplanted into the mice. All animal experiments were executed by Wuhan University Animal Care Facility and National Institutions of Health Guidelines.

### *In vivo* Study

When the tumor size reached about 100 mm^3^, treatment was commenced. Mice were randomly separated into six groups (5 mice per group) and treated as follows: (i) Control (100 μL PBS), (ii) FePd@CNTs (i.v., injection; 100 μL, 200 μg/mL), (iii) RT (6 Gy), (iv) FePd NPs (i.v., injection; 100 μL, 200 μg/mL) +RT (6 Gy), and (v) FePd@CNTs (i.v., injection; 100 μL, 200 μg/mL) +RT (6 Gy). After 24 h for injection, mice in Group iii, iv, and v were treated by RT (6 Gy) using Small Animal Irradiator (PXI X-RAD 225 Cx, North Branford, CT, United States). For every 2 days, the weight of mice and the volume of tumors were recorded. The formula (length × width^2^)/2 (Von Kalle et al., 1986) was used to calculate tumor volume (V). All mice were killed after 14 days of treatment. Main organs (hearts, livers, spleens, lungs, and kidneys) were extracted, fixed in 4% paraformaldehyde. The fixed organs were sliced and stained with hematoxylin and eosin (H&E) after the paraffin embedding. The tumor tissues were also fixed in 4% paraformaldehyde, embeded into paraffin and sliced into tissue sections. Thereafter, the sections were stained with Ki-67, dihydroethidium (DHE), and terminal deoxynucleotidyl (TUNEL) are examined by an inverted fluorescence microscope (Olympus IX 73 DP 80, Japan).

### Pharmacokinetics and Biodistribution

The pharmacokinetics were determined in mice (*n* = 5). FePd NPs and FePd@CNTs (200 μg/mL, 100 μL) were injected into healthy BALB/c mice via tail vein, followed by collecting blood samples at 0.5, 1, 2, 4, 8, 12, 24, and 48 h. To study the biodistribution, the mice were administrated with FePd NPs and FePd@CNTs (200 μg/mL, 100 μL). After 24 h, the mice were sacrificed and main organ were harvested which were later dissolved in concentrated HNO_3_. The concentrations of Pd in blood samples were obtained using ICP-MS.

### Statistical Analysis

GraphPad Prism 8.0 software was used for statistical analyses. All measurements are presented as the mean ± standard deviation (SD). A one-way ANOVA followed by the post-Tukey comparison test was used to determine the differences between the groups. A *p*-value < 0.05 was considered statistically significant.

## Results and Discussion

### Preparation and Characterization of FePd@CNTs

FePd@CNTs nanocomposites were successfully synthesized via a convenient one-pod reaction. In brief, Fe(acac)_3_, Na_2_PdCl_4_, oleylamine and oleic acid were successively dissolved in anhydrous ethanol with magnetic stirring, followed by adding dropwise a mixture of carbon nanotubes and PVP dispersed in anhydrous ethanol. Then, excess ethanol solution of NaBH_4_ was added dropwise into the mixture. The mixture was stirred for 2 h at 40°C. Finally, product was separated by centrifugation and dried in a vacuum oven to obtain final product FePd@CNTs.

**FIGURE 1:**
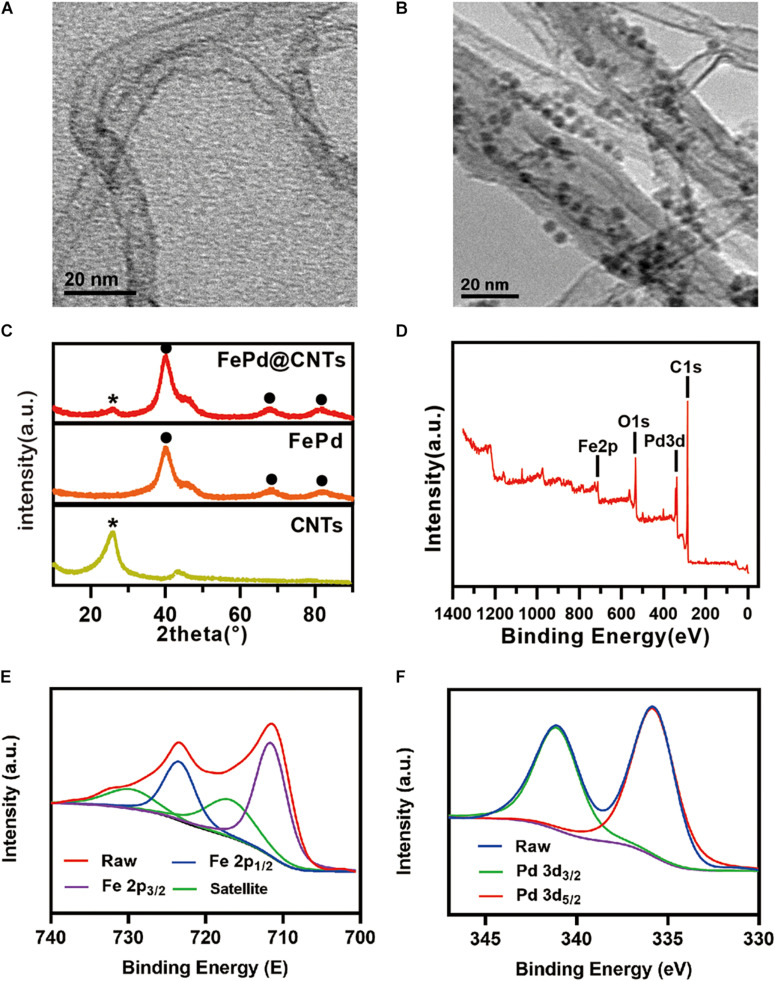
Characterization **(A,B)** TEM images of CNTs and FePd@CNTs. **(C)** XRD patterns of CNTs, FePd NPs, and FePd@CNTs. **(D)** XPS analysis of FePd@CNTs. **(E,F)** XPS spectra of Fe 2p and Pd 3d orbits.

The size and morphology of FePd, carbon nanotubes and FePd@CNTs were observed by TEM. As observed in Supplementary Figures 1A,B, FePd is uniform of 3–4 nm in diameter. As shown in Figures 1A,B, quantities of nanodots with diameter of 3–4 nm were uniformly attached on the surface of carbon nanotubes, indicating the FePd NPs were successfully decorated on the surface of carbon nanotubes via our new method, which could also be observed in Supplementary Figure 1C. The energy dispersive spectroscopy (EDS) analysis indicates the obtained nanomaterial is composed of C, Fe, and Pd elements with the element ratio of 50.1:49.9 (iron: palladium). The X-ray powder diffraction (XRD) pattern in Figure 1C displayed a representative (002) peak of the carbon nanotubes phase at 2θ = 26.4°, and peaks corresponding to the (111), (220), and (311) lattice planes of Pd phase at 2θ = 40.1°, 68.1°, and 60.1°. The X-ray photoelectron spectroscopy (XPS) spectra in Figure 1D We can see obvious characteristic peaks of C 1 s at 284.80 eV, Pd 3d at 335.67 eV, O 1 s at 532.03 eV, and Fe 2p at 711.26 eV. Both the high resolution of spectra of Fe 2p and Pd 3d orbits were shown in Figures 1E,F, respectively. The zeta potentials of FePd, CNT and FePd@CNT were 19.3, 29.0, and 32.3 mV, respectively (Supplementary Figure 3). These results demonstrated that FePd NPs have been successfully decorated on CNTs.

### *In vitro* Biocompatibility Assay

**FIGURE 2:**
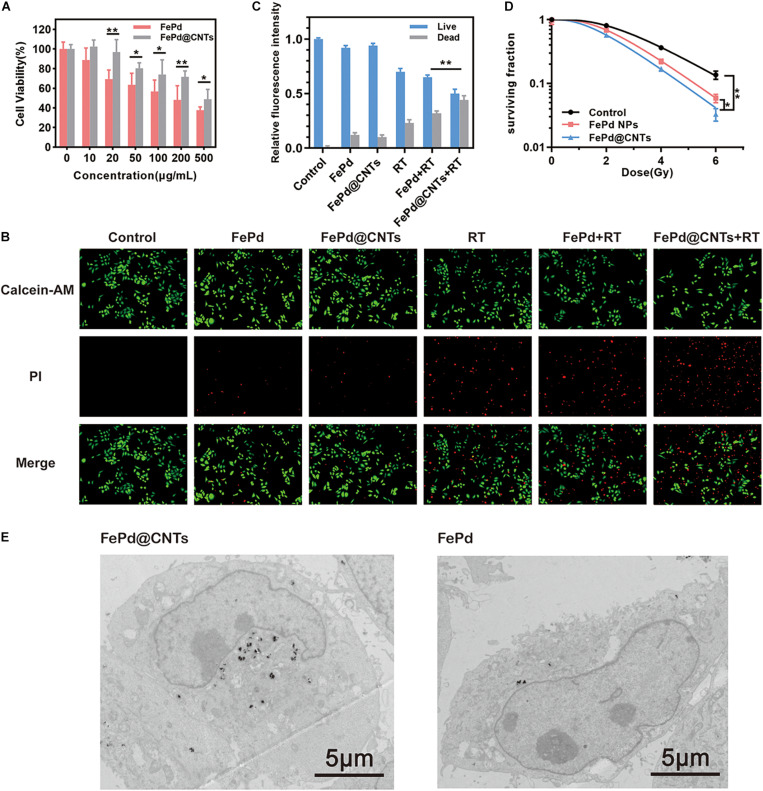
*In vitro* study **(A)** Cytotoxicity of various concentration of FePd and FePd@CNTs for MCF-7 cells. **(B)** Live/dead staining of MCF-7 cells in different groups. **(C)** The relative fluorescence intensity of Live/dead cells, expressed as fold intensity of live cells in control group (value = 1). **(D)** Survival curves of MCF-7 cells in three groups. (*p < 0.05, **p < 0.01). **(E)** TEM images of MCF-7 cells incubated with FePd@CNTs and FePd NPs.

Biocompatibility of FePd@CNTs was evaluated via the CCK 8 assay on MCF-7 cells (breast cancer). FePd NPs was set as control group. Cell toxicity of FePd@CNTs at different concentrations (0, 10, 20, 50, 100, 200, and 500 μg/mL) was investigated. As demonstrated in Figure 2A, we found that the cell viability of MCF-7 cells in group FePd@CNTs is superior to group FePd at all concentrations. The cell viability of MCF-7 cells treated with FePd@CNTs was maintained at about 72% with a high concentration of 200 μg/mL. The IC 50 value of FePd@CNTs was calculated to be 482.5 μg/mL. All these results above suggested that FePd@CNTs possessed good biocompatibility.

### The Radiosensitization Effect *in vitro*

Whether the ratio of iron to palladium affect the therapy effect arose our interests. Here, we synthesized nanomaterials with various proportions of iron and palladium. Fe_3_Pd@CNTs represents the ratio of iron to palladium is 3 for instance. Next, the radiosensitization effect of these nanomaterials was assessed using CCK 8 assay. As shown in Supplementary Figure 4, viability of cells treated with FePd@CNTs, Fe_3_Pd@CNTs or FePd_3_@CNTs showed no significant difference. Moreover, the radiosensitization ability may not depend on the ratio either since no obvious distinction was found in the latter three groups. For that reason, we used FePd@CNTs for the following study.

MCF-7 cell survival rates were further analyzed using a calcein AM-PI live-dead staining assay (Figure 2B). The cells were divided into six groups and treated with: (i) Control (100 μL PBS), (ii) FePd NPs (i.v., injection; 100 μL, 200 μg/mL), (iii) FePd@CNTs (i.v., injection; 100 μL, 200 μg/mL), (iv) RT (6 Gy), (v) FePd (i.v., injection; 100 μL, 200 μg/mL) +RT (6 Gy), and (vi) FePd@CNTs (i.v., injection; 100 μL, 200 μg/mL) +RT (6 Gy). The fluorescence intensity of dead cells in FePd@CNTs+RT group is obviously higher than FePd+RT groups, indicating FePd@CNTs+RT might cause more disastrous cell damage. The quantitative relative fluorescence intensity histogram of live/dead cells was shown in Figure 2C. The red fluorescence intensity of FePd group is slightly higher than that of FePd@CNTs group, indicating weaker cytotoxicity of FePd@CNTs than FePd NPs. Compared with FePd group and FePd@CNTs group, the fluorescence of dead cells in FePd RT and FePd@CNTs + RT groups are about two and three times stronger, respectively. Particularly, the red fluorescence intensity of FePd@CNTs+RT group is about 4.8 times greater than FePd@CNTs group. The results indicate that FePd@CNTs displayed an improved radiosensitization effect, which is because CNTs could deliver more FePd NPs into cells, compared to FePd NPs alone.

The level of DSBs in tumorous cells is an important index in assessing antitumor effect. Herein, expression of γ-H_2_AX in various treatment groups were evaluated as shown in Supplementary Figure 6A. The group treated with FePd, FePd@CNTs, and RT exhibited low DSBs level. The FePd + RT group showed a moderate cell cytotoxicity which was due to the radiosensitization effect of FePd. However, the group pretreated with FePd@CNTs prior to RT demonstrated a high level of DNA damage, which is consistent with the fluorescence intensity analysis result in Supplementary Figure 6B. Next, the ROS content in each group was assessed using fluorescence probe DCFH-DA (Supplementary Figure 6C). After incubation with FePd@CNTs, RT was found to produce the highest amount of ROS in cells among all groups.

### Colony Formation Assay

Colony formation assay was performed to assess enhancement of radiosensitivity by FePd NPs and FePd@CNTs. MCF-7 cells were incubated with various processing methods for 24 h, and then given X-ray doses of 0, 2, 4, or 6 Gy. Later, the colony counts of two groups were counted and calculated. A multi-target single-hit model was used to fit the cell survival curve, radiobiological parameters such as the mean lethal dose (D_0_), the quasi-threshold dose (D_*q*_) and the survival fraction at 2 Gy (SF_2_) were calculated. As shown in Figure 2D, following treatment with 200 μg/mL FePd or FePd@CNTs, the MCF-7 cell survival curves shifted to the left, the shoulder areas were diminished, and the steepness of the curves increased, suggesting that both FePd and FePd@CNTs enhanced the radiation sensitivity of MCF-7 cells. The radiobiological parameters including D_0_ (mean lethal dose or final slope), D_*q*_ (quasi-threshold dose) and SF_2_ (surviving fraction of 2 Gy) and SER (sensitizing enhancement ratio) corresponding each group were listed in Table 1. The SER value of group a was calculated as followed:

$$SER=\frac{D_{0,control}}{D_{0,a}}$$

**TABLE 1:** Radiobiological parameters of cell survival curve.

	D_0_	Dq	SF_2_	SER
Control	1.75	2.54	0.81	–
FePd	1.42	2.02	0.69	1.23
FePd@CNTs	1.39	1.59	0.57	1.26

The SER value of FePd group and FePd@CNTs group are 1.23 and 1.26, indicating that FePd@CNTs have a better radiosensitizing effect than FePd NPs.

Moreover, to further confirm the effect of FePd@CNTs on radiosensitization, the result of colony formation was also fit using LQ model method as shown in Supplementary Figure 3. The relative parameters including α and β were demonstrated in Supplementary Table 1. Consistent with the result of multi-target single-hit model, the group pretreated with FePd@CNTs to X rays possess the highest value of α/β, which is 2.264, suggesting a superior radiosensitization ability over FePd.

### Cellular Uptake

The cellular uptake and intracellular distribution of FePd NPs and FePd@CNTs in MCF-7 cells were obtained by TEM. After incubating with FePd@CNTs or FePd NPs for 24 h, there were highly electron-dense material accumulation being observed in TEM imagines (Figure 2E) of MCF-7 cells, indicating these nanomaterials were “eaten” by cells. Moreover, it can be seen that MCF-7 cells took more FePd@CNTs than FePd NPs, proving that FePd@CNTs could be assimilated by cancer cells with higher efficiency, which is consistent with our assumption. Moreover, it is further confirmed that the cellular uptakes in quantity after the incubation of MCF-7 cells with FePd or FePd@CNTs. As shown in Supplementary Figure 5, FePd@CNTs were taken up higher than FePd in MCF-7 cells. Hence, carbon nanotubes attributed to the cellular uptakes.

### *In vivo* Synergistic Radiotherapy

**FIGURE 3:**
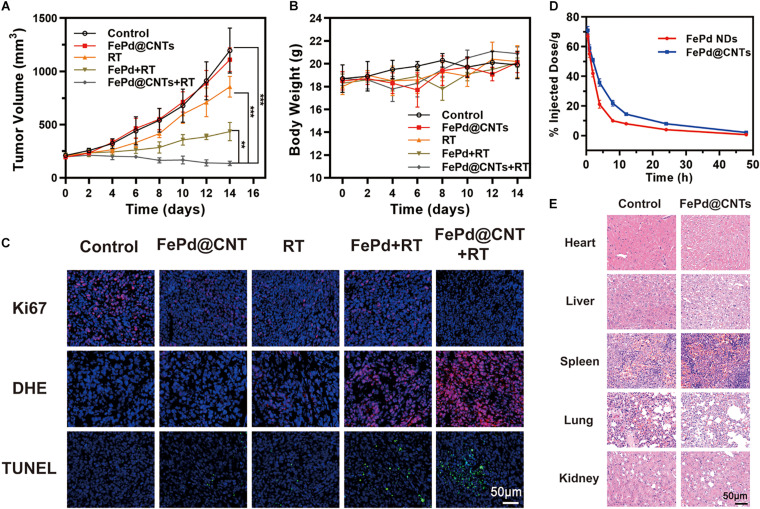
*In vivo* study **(A)** Tumor volume change curve of mice. **(B)** Body weight change curve of mice. **(C)** Ki67, DHE and TUNEL staining of tumor. **(D)** Relative concentrations of FePd NPs and FePd@CNTs at different times after administration. **(E)** H&E staining of main organs of mice. (**p < 0.01, ***p < 0.005).

The therapeutic potential of FePd NPs and FePd@CNTs in the BALB/c mice was conducted to investigate the *in vivo* antitumor effects of FePd@CNTs. Mice were treated by five various methods as follows: (i) Control (100 μL PBS), (ii) FePd@CNTs (i.v., injection; 100 μL, 200 μg/mL), (iii) RT (6 Gy), (iv) FePd NPs (i.v., injection; 100 μL, 200 μg/mL) +RT (6 Gy), and (v) FePd@CNTs (i.v., injection; 100 μL, 200 μg/mL) +RT (6 Gy). As described in Figure 3A, the control group exhibits a rapid increase of tumor volumes. Compared with the control group, there is no obvious influence on tumor growth when treated with FePd@CNTs alone, suggesting that FePd@CNTs have low toxicity *in vivo*. The tumor treated by radiotherapy without nanodrugs exhibited slightly inhibition of tumor growth. However the FePd NPs+RT group showed a more obvious effect to inhibit the tumor growth. Moreover, the FePd@CNTs+RT group even exhibited a decrease of tumor volume. The Tumor Growth Inhibition value (TGI) of FePd@CNTs+RT group is 88.6%, is much higher than FePd@CNTs group (7.2%), RT group (28.4%) and FePd+RT group (63.6%). Thus, it can be concluded that FePd@CNTs have a great radiotherapy enhancement effect. In addition, the body weight change curve of mice (Figure 3B) showed that the body weight of mice had no obvious change in different group.

Afterward, Ki67, DHE and TUNEL assays immunofluorescence staining of tumor sections were performed to appraise the tumor tissue dilapidate (Figure 3C). Ki-67 is a biomarker of cell proliferation with red fluorescent represents cells with proliferating ability. The fluorescence images of Ki-67 showed that cells of control group are the most proliferative. The Ki-67 fluorescence intensity of FePd@CNTs+RT group is lower FePd+RT group, suggesting that FePd@CNTs+RT has a significant effect on cell proliferation inhibition. The ROS generation in tumor tissues was valued by DHE staining. Compared with Control, FePd and RT group whose content of ROS (red) are deficient, the ROS level exhibited an obvious increase in FePd+RT group. Furthermore, tumors treated with FePd@CNTs+RT have the maximal ROS content, indicating that the combination of FePd@CNTs and radiation could distinctly induce ROS production. TUNEL assay was performed to exhibit apoptosis of tumors. In the control group, only a few apoptotic cells were observed. The cell apoptosis in FePd@CNTs group and RT group are both at a low level. The FePd+RT group and FePd@CNTs+RT group, however, exhibit obvious apoptosis while the latter has the most extensive apoptosis, indicating that FePd@CNTs combined with radiation could induce the most significant cell apoptosis. As has been demonstrated, compared with FePd, FePd@CNTs showed more significant cellular uptakes and more obvious tumor accumulation, which was attributed to the carrier carbon nanotubes. Meanwhile, FePd@CNTs+RT groups exhibited the highest density of ROS fluorescence intensity both *in vitro* and *in vivo* among all groups. Therefore, it can be concluded that FePd@CNTs in tumor may induce apoptosis via generation and accumulation of ROS after irradiation, thus inhibiting cell proliferation.

We further studied the pharmacokinetic of FePd and FePd@CNTs on BALB/c mice. FePd@CNTs exhibited a prolonged circulation time than FePd as shown in Figure 3D, which was attributed to the larger size of FePd@CNTs and better biocompatibility of FePd@CNTs. Moreover, as demonstrated in Supplementary Figure 7, the spleen was shown to possess the highest uptake of nanoparticles at 24 h post-injection. Besides, FePd@CNT showed much better tumor accumulation than FePd. Therefore, the retention and accumulation of FePd@CNTs in tumor site can be elevated to enhance the effect of radiotherapy.

### Safety Evaluation

To evaluate the *in vivo* safety of the FePd@CNTs in mice, major organs (heart, liver, spleen, lung, and kidney) were collected from mice in group control and FePd@CNTs and hematoxylin and eosin (H&E) staining was performed. As shown in Figure 3E, neither obvious damage nor lesions were observed in group FePd@CNTs, indicating that FePd@CNTs have no significant toxicity *in vivo*.

## Conclusion

In this work, FePd decorated carbon nanotubes were syntheses via chemical reduction method. Such a carbon nanotube carrier enhances the efficiency in drug delivery due to their ability to cross cell membranes as well as prolonged the circulation time *in vivo*. Moreover, FePd@CNTs induced cell apoptosis through generating quantities of intracellular ROS. Both *in vitro* and *in vivo* experiments exhibited significantly radiosensitizing effect of FePd@CNTs with negligible toxicity to normal tissues. Overall, the results confirmed that FePd@CNTs as a potential sensitizer for radiotherapy. Further studies are needed to elucidate the specific detailed mechanism in radiosensitizing.

## Data Availability Statement

The original contributions presented in the study are included in the article/Supplementary Material, further inquiries can be directed to the corresponding author/s.

## Ethics Statement

The animal study was reviewed and approved by the Wuhan University Animal Care Facility and National Institutions of Health Guidelines.

## Author Contributions

All authors listed have made a substantial, direct and intellectual contribution to the work, and approved it for publication.

## Conflict of Interest

The authors declare that the research was conducted in the absence of any commercial or financial relationships that could be construed as a potential conflict of interest.
