# Supplementary material for: Iron-Palladium Decorated Carbon Nanotubes Achieve Radiosensitization via Reactive Oxygen Species Burst
Source: Front Bioeng Biotechnol. 2021 May 21;9:683363. doi: 10.3389/fbioe.2021.683363 (PMC8176102; doi:10.3389/fbioe.2021.683363)
Supplement: Supplementary file 1 [file Data_Sheet_1.docx]

Supporting information

**Iron-palladium decorated carbon nanotubes achieve radio-sensitization via reactive oxygen species burst**

Shengnan Yang^a1^, Yiling Yang^b1^, Yi Yang^a^, Xiangya Zhao^a^, Qian Wang^a^, Bing Li^a^, Ling Dong^a^, Rui Tian^a*^ and Zhirong Bao^c*^

^a^ Department of Geriatric Medicine, The First Affiliated Hospital of Zhengzhou University, Zhengzhou,450052, China

^b^ Department of Ultrasound, The First Affiliated Hospital, Zhengzhou University, Zhengzhou,450052, China

^c^ Department of Radiation and Medical Oncology, Hubei Key Laboratory of Tumor Biological Behaviors, Hubei Cancer Clinical Study Center, Zhongnan Hospital of Wuhan University, Wuhan 430071, China

Correspondence to:

Rui Tian

Department of Geriatric Medicine, The First Affiliated Hospital of Zhengzhou University, Zhengzhou, 450052, China

fcctianr@zzu.edu.cn

Zhirong Bao

Department of Radiation and Medical Oncology, Hubei Key Laboratory of Tumor Biological Behaviors, Hubei Cancer Clinical Study Center, Zhongnan Hospital of Wuhan University, Wuhan 430071, China

bao_zhirong@163.com


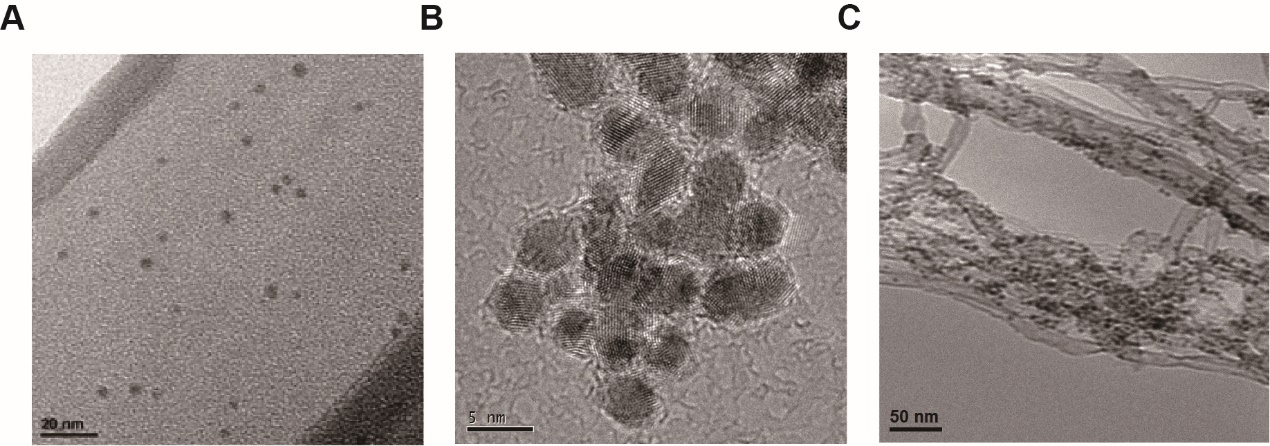


**Figure S1**. TEM images of (A) FePd NPs, (B) high-resolution of FePd NPs and (C) large-scale of FePd@CNTs.


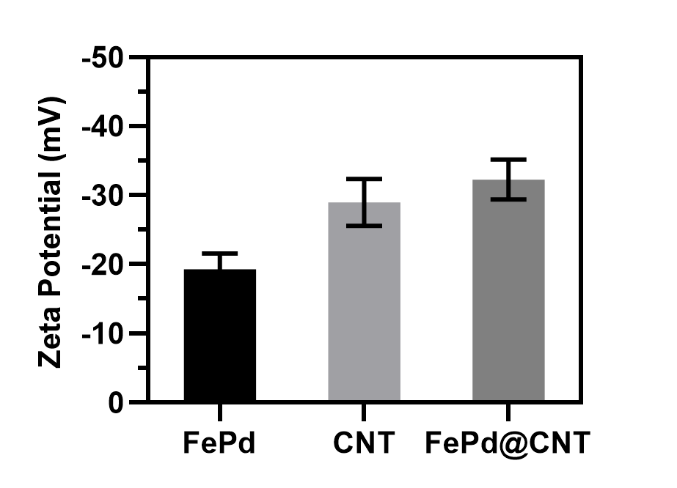


**Figure S2**. Zeta potential of FePd, CNT and FePd@CNT.


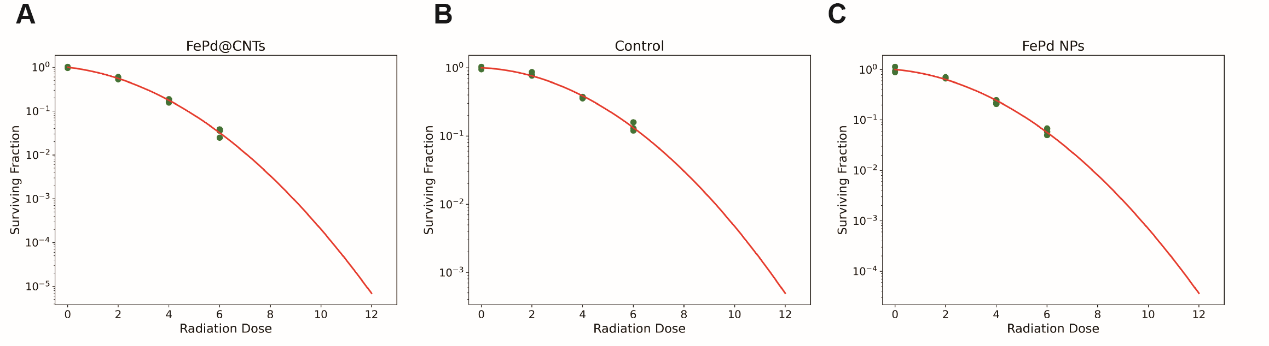


**Figure S3**. Colony formation analysis using LQ-model. (A) Control. (B) Pretreated with FePd. (C) Pretreated with FePd@CNTs.

**Table S1.** The α and β value in each group.

|  | Control | FePd | FePd@CNTs |
| --- | --- | --- | --- |
| α | 0.039 | 0.104 | 0.157 |
| β | 0.049 | 0.062 | 0.069 |
| α/β | 0.794 | 1.678 | 2.264 |


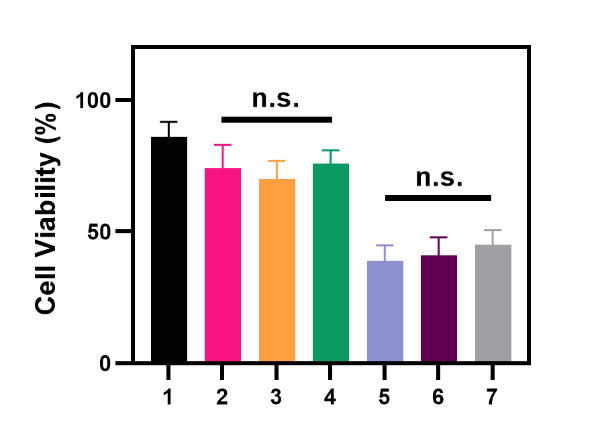


**Figure S4**. Cell viability after various treatments (1: RT; 2: FePd@CNTs; 3: FePd_3_@CNTs; 4: Fe_3_Pd@CNTs; 5: FePd@CNTs+RT; 6: FePd_3_@CNTs+RT; 7: Fe_3_Pd@CNTs+RT).


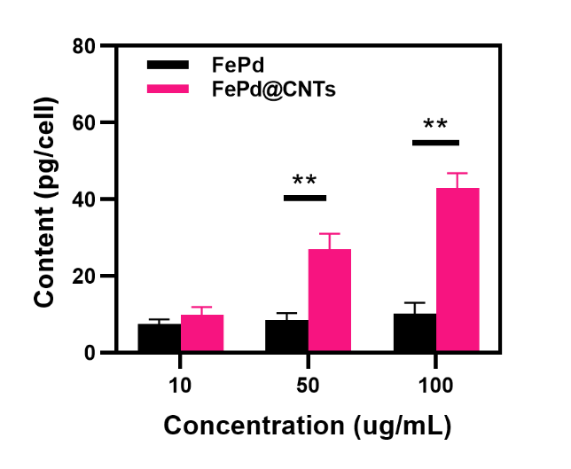


**Figure S5** Pd content in MCF-7 cells after treated with FePd or FePd@CNTs of various concentrations.


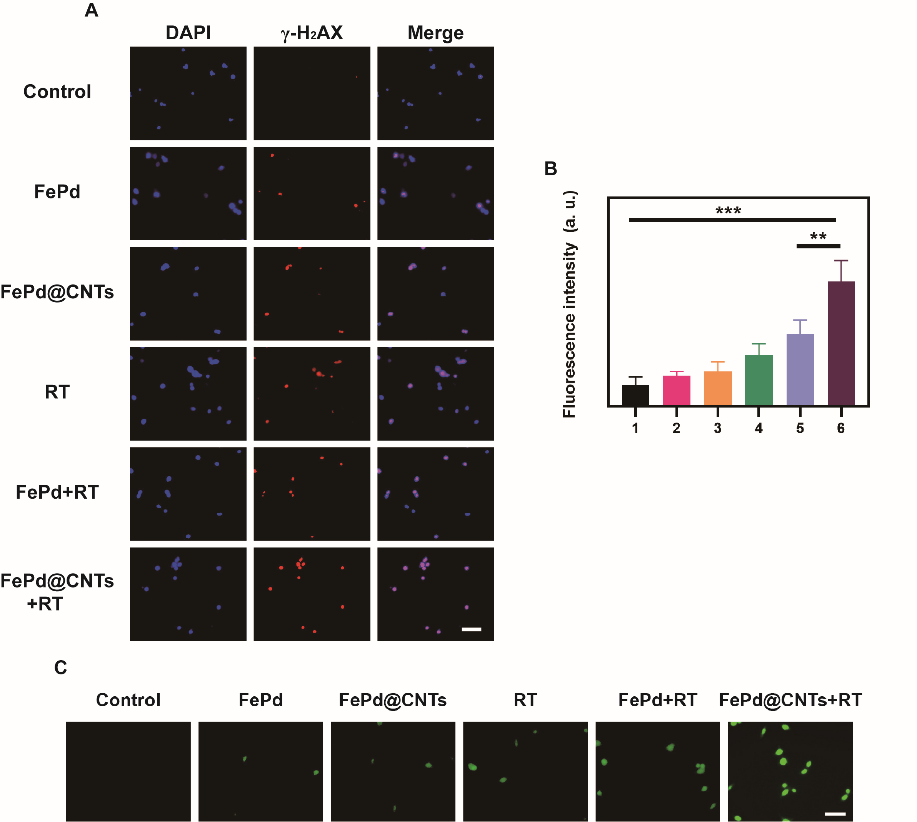


**Figure S6**. DSBs analysis. (A) Images of γ-H_2_AX staining in each group and (B) corresponding fluorescence intensity (1: RT; 2: FePd@CNTs; 3: FePd_3_@CNTs; 4: Fe_3_Pd@CNTs; 5: FePd@CNTs+RT; 6: FePd_3_@CNTs+RT; 7: Fe_3_Pd@CNTs+RT). (C) CLSM images of MCF-7 staining with DCFH-DA after various treatments.


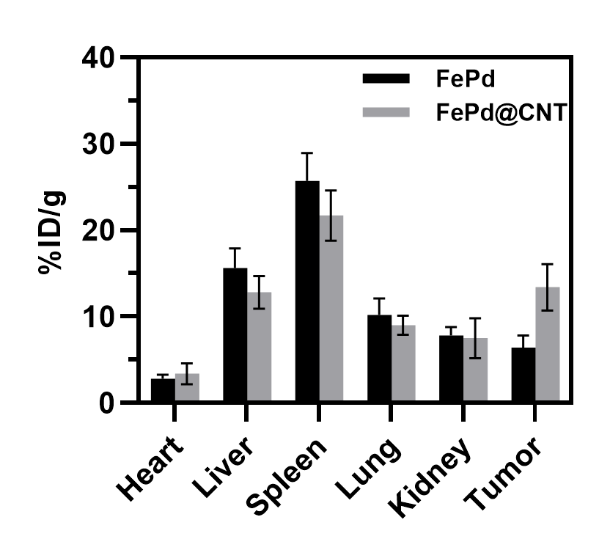


**Figure S7.** Biodistribution of FePd and FePd@CNT 24 h post i.v. injection.
